# Analysis with respect to instrumental variables for the exploration of microarray data structures

**DOI:** 10.1186/1471-2105-7-422

**Published:** 2006-09-29

**Authors:** Florent Baty, Michaël Facompré, Jan Wiegand, Joseph Schwager, Martin H Brutsche

**Affiliations:** 1 Pulmonary Gene Research, Universitätsspital Basel, Petersgraben 4, 4031 Basel, Switzerland; 2 DSM Nutritional Products, Human Nutrition and Health, Wurmisweg 576, CH-4303 Kaiseraugst, Switzerland

## Abstract

**Background:**

Evaluating the importance of the different sources of variations is essential in microarray data experiments. Complex experimental designs generally include various factors structuring the data which should be taken into account. The objective of these experiments is the exploration of some given factors while controlling other factors.

**Results:**

We present here a family of methods, the analyses with respect to instrumental variables, which can be easily applied to the particular case of microarray data. An illustrative example of analysis with instrumental variables is given in the case of microarray data investigating the effect of beverage intake on peripheral blood gene expression. This approach is compared to an ANOVA-based gene-by-gene statistical method.

**Conclusion:**

Instrumental variables analyses provide a simple way to control several sources of variation in a multivariate analysis of microarray data. Due to their flexibility, these methods can be associated with a large range of ordination techniques combined with one or several qualitative and/or quantitative descriptive variables.

## Background

Microarray experiments essentially yield highly multivariate data. The number of variables measured in such data is generally far greater than the number of samples and numerous specific statistical approaches have been proposed. In this context, ordination methods proved to be powerful exploratory tools.

Principal component analysis (PCA) and correspondence analysis (CA) are two dimensionality reduction techniques commonly applied in this area of microarray analysis [1,2]. In an unsupervised fashion, these techniques aim to summarize trends present in high-dimensional datasets.

Besides the variables of direct interest (gene expression levels), one or several qualitative variables are sometimes used to describe features of the experimental design. In a clinical context, variables describing the phenotypic structures of a population are typically involved (e.g. "healthy controls" vs. "patients"). Several other variables can also be taken into account including information about temporal, treatment, individual effects, etc. Technical information can also be described. For example, laboratory effect and batch effect not rarely represent an important source of data variation. Overall, the descriptive variables can be classified into two categories: those which are relevant to the analysis, and those whose influence should be removed from the analysis.

Basic ordination methods like PCA or CA extract information present in a dataset independently of *a priori *experimental structures. On the other hand, one may wish to take into consideration the effect of different variables controlled in the experimental design. Constrained ordination methods were developed for this purpose. A variety of methods has been developed mainly in the context of environmental science but only exceptionally have been applied to genomics data. These methods include (partial) canonical correspondence analysis (CCA) [3,4], redundancy analysis (RDA) [5] and principal component analysis on instrumental variables (PCAIV) [6]. The objective of these methods is to link a table of variables of interest with a table of discriminative variables. Kenkel and colleagues [7] provided an interesting comparative overview of some of these techniques. In their review, RDA is described as the canonical form of PCA and CCA as the canonical form of CA. RDA and CCA can actually be described as two particular cases of analysis with respect instrumental variables. Because these techniques take explicitly a grouping information into account, they can be considered as the supervised counterpart of classical ordination methods.

In this paper, we introduce the analyses with respect to instrumental variables, and more specifically, two particular cases: within- and between-group analyses. We will show how these approaches can be applied to explore experimental data structures in order to remove some undesirable effects while focusing on other particular effects. An example with microarray data measuring the effect of beverage intake on individuals over time is given. Results are compared with those obtained with an alternative gene-by-gene analysis based on the fit of a linear regression model. Advantages and limitations are discussed.

## Results

### Analyses with respect to instrumental variables

Theoretical aspects about instrumental variable techniques are detailed elsewhere [8-12]. In brief, instrumental variable methods aim to match a statistical triplet (**Y**, **D**_*n*_, **D**_*m*_) with a matrix of descriptive variables **X**. **Y **(*n *× *m*) is the table to be analyzed, **D**_*n *_and **D**_*m *_are respectively the row and column weight diagonal matrices, and **X **(*n *× *p*) a matrix including descriptive variables which can be either qualitative, quantitative or both. In such analyses, **X **and **Y **play a dissymmetric role. **Y **contains the variables of direct interest, whereas **X **contains structural information about samples. The information of **X **is used to constrain the analysis of **Y**.

Using a regression terminology, **Y **contains the dependent variables and **X **the independent variables. Each variable from **Y **is predicted based on variables from **X **using multiple linear regression. Models are in the form of:

**Y **= *α *+ *β***X **+ *ε*

Model estimates are combined in a third table **Ŷ **from which principal components are calculated. From a geometrical point of view, these *p *models are obtained by projecting variables from **Y **on the sub-space formed by the descriptive variables in **X**. Principal components under constraints maximize the sum of squared correlations with variables from **Y**.

Two categories of methods on instrumental variables can be distinguished: the direct and orthogonal methods. Direct methods take effects of descriptive variables positively, whereas orthogonal ones take these effects negatively into account. In the latter case, the analysis is performed on the model's residuals. It is generally used when one wishes to remove some unexpected effects. Finally, it is possible to combine positive effects with negative effects in the same analysis, which makes it possible to simultaneously analyze a given effect by removing another effect. In such a case, the effect of the conditional variables is first removed from the data, then a constrained analysis is performed on the residual matrix.

### Between-group/within-group analyses

**Figure 1:**
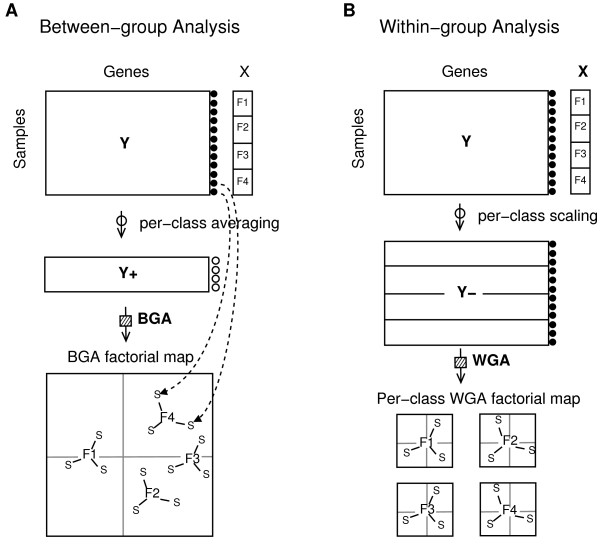
**Between/within-group analysis**. Schematic representation of BGA and WGA procedures adapted from [11]. For BGA (panel A), the analysis of table **Y **is initially performed on the per-class sample average table **Y**+ and every sample is projected on the factorial map (2 first principal axes). For WGA (panel B), samples in **Y **are scaled by dividing them by the per-class means, and the analysis is performed on the scaled table **Y**-. The per-class factorial map of WGA (2 first principal axes) is centered around 0.

Between-group and within-group analyses are two particular cases of instrumental variable methods where a single qualitative variable is accounted for. The use of between-group and within-group analyses enables to take respectively positive and negative constraints into account in a very simple and flexible way. Between-group analyses (BGA) are performed in two steps. The table containing the variables of interest is transformed according to the constraint, then a single-table ordination method is applied. For example, a between-group correspondence analysis of the triplet (**Y**, **D**_*n*_, **D**_*m*_) is obtained by doing a correspondence analysis on the triplet (**Y**+, **D**_*n*_, **D**_*k*_), where **Y**+ is the table of per-class means of **Y **and **D**_*k *_the diagonal matrix of class weights. Geometrically, the per-class centers of gravity are projected on the BGA discriminating axes and the whole set of samples is projected as supplementary rows (Figure 1, panel A). This procedure provides the best linear combination of variables which maximizes the between-group variance. Culhane and colleagues [13] demonstrated the efficiency of BGA in microarray data, especially when associated with CA.

Within-group analyses (WGA) are complementary to BGA. Similarly to BGA, WGA can be associated with any single-table ordination method. In WGA, the analysis is focused on the table of residuals after scaling the data by the per-class means (Figure 1, panel B). This procedure eliminates the effect due to the constraint. It is carried out when one wishes to explore the structures of a table independently of an undesirable effect.

BGA can be used to measure the structural contribution (in terms of inertia) of different qualitative variables in a microarray dataset. WGA can be used to get rid of a specific unexpected effect. As proposed in this manuscript, BGA and WGA can also be combined to take both positive and negative constraints into account.

### Example of structure exploration in microarray data

In this example, microarray experiments were performed in order to analyze the influence of beverage intake over time in blood gene expression. Six healthy volunteers participated in this randomized controlled cross-over experiment. On 4 independent days they had 4 different beverages (350 mL each: grape juice, red wine, 40 g diluted ethanol, water). The diluted ethanol, refered below as "alcohol", was calibrated to reach the same total amount and concentration of alcohol as red wine. Blood samples were taken at baseline, 1, 2, 4, 12 h after the drink together with standardized nutrition. Messenger RNA of 120 peripheral blood lymphocyte samples were hybridized on Affymetrix microarrays HGU133A including 22283 genes (raw files have been deposited in NCBIs Gene Expression Omnibus (GEO), and are accessible through GEO Series accession number GSE3846). The data quality was checked, and microarrays with poor quality were removed from the dataset. A total of 108 microarrays were finally included in the analysis (supplementary material is available as an online repository [14]). The dataset was normalized using RMA [15]. Three sources of variations were examined: "individual", "time" and "beverage".

The data is structured in two tables:

• **Y **is the table of gene expression intensities (108 samples × 22283 genes)

• **X **is the table with 3 descriptive variables giving a structural information among samples (108 samples × 3 factors)

The objective of the data analysis is to couple these two tables, the analysis of **Y **being constrained by the information of **X**.

**Figure 2:**
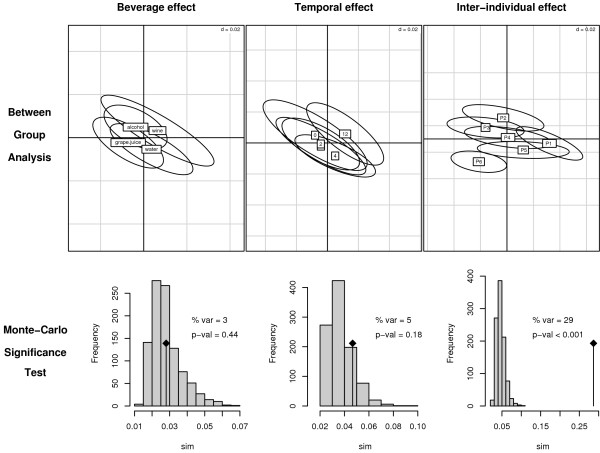
**Dataset sources of variations**. Decomposition of the dataset variability according to three sources of variations: "individual", "time" and "beverage". Ellipsoids representing the distribution of samples around the per-class centers of gravity are plotted on the factorial map of BGA (2 first discriminating axes). For each BGA, a Monte-Carlo permutation test is performed to assess the significance of the structures modelled in the analysis. The histograms show the distribution of 999 simulated values of the randomization test for BGA together with the observed value. Sim: ratio of between-class and total inertia.

In a first step, the effect of the constraints was studied one by one. The percentage of variability attributed to each of the three sources of variation was explored using between-group correspondence analysis (BGA combined with CA) (Figure 2). This parameter corresponds to the ratio of the total inertia measured in BGA on the total inertia measured in CA. Inter-individual variability ("individual") is the main source of variation since it explains 29% of the whole variability. Only 5% of the variability is explained by the temporal variation ("time"), and one may simply note that the first BGA discriminant axis tends to describe a circadian variation ("12 h"-ellipsoid is slightly shifted out compared to the other time points). Finally, no clear effect was visible as far as the beverage is concerned. It is likely that this effect is hidden by some more preponderant effects, including the individual and temporal effects.

When exploring the effect of beverage intake at all time points except baseline (time points 1, 2, 4 and 12 h), no significant beverage effect could be found when using regular BGA (p-val = 0.4). However, the effect of beverage can be studied independently of the "individual" effect. For this purpose, correspondence analysis with respect to instrumental variables (CAIV) was applied, taking the "individual" effect negatively and the "beverage" effect positively. Due to the removal of the "individual" effect, the effect of "beverage" became significant (p-val = 0.04). In addition, CAIV taking the "individual" effect negatively and the "time" effect positively, results in a significant time-course pattern as well (p-val = 0.02).

**Figure 3:**
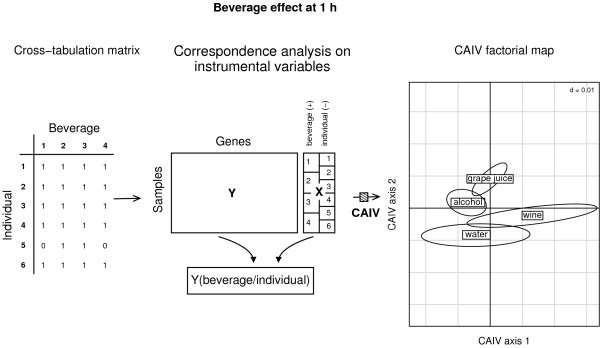
**Correspondence analysis with respect to instrumental variables**. The between-beverage analysis applied to the data where the "individual" effect has been removed, shows a structure associated with the effect of red wine on the first discriminating axis. Two factors were successively included in the analysis: "individual" effect (6 modalities) and "beverage" effect (4 modalities). The "beverage" effect was taken positively while the "individual" effect was controlled. The analysis is focused on the 1 h-time point.

CAIV is equivalent to a conditional between-group correspondence analysis (Figure 3): the "beverage" effect was analysed conditionally to the "individual" effect (*A*/*B*). Let define Y the initial table, X1 the negative variable ("individual") and X2 the positive ("beverage"). Two successive procedures are needed to perform a CAIV. The first procedure consists in removing the undesirable effect using a within-group analysis (one single qualitative variable). The second procedure consists in analysing specifically the constraint of interest by using a between-group analysis (one single qualitative variable). Using an R syntax, CAIV is simply obtained by:

library(ade4)

ca <- dudi.coa(Y,scannf=F)

wg <- within(ca,X1,scannf=F)

bgwg <- between(wg,X2,scannf=F)

s.class(bgwg$ls,X2)

In the general case where the number of positive and negative constraints is higher than one, functions between and within must be respectively replaced by pcaiv and pcaivortho.

Genes associated with the different drinks were identified based on the loadings obtained after CAIV. The orthogonal projection of genes on the vectors of each class centroids is used to determine the association of genes accoffding to the classes. Discriminating genes are sorted and up/down-regulated genes are extracted. Although, a detailed description of the list of genes obtained by this analysis is out of the scope of this paper, several key genes known to be associated with the consumption of alcohol but also with mechanisms of action of compounds present in red wine were identified.

The results indicate a clear toxic effect of "alcohol" in the early time points. For example, at time "1 h", within the list of the 100 most up-regulated genes, there was a significant enrichment of genes associated to the Gene Ontology categories "response to stimulus", "response to stress", "immune response" and "apoptosis" (GO:0009607, GO:0006955: p-val < 0.001; GO:0006954: p-val = 0.02; GO:0006950: p-val = 0.003; p-val < 0.001; GO:0006915: p-val < 0.001). This toxic effect, as measured by the enrichment of genes in these GO categories, was maximal at time "1 h", and it persisted at time "2 h" (all above mentionned GO categories were significantly enriched) and "4 h" (all above mentionned GO categories except GO:0006954 and GO:0006950 were significantly enriched). Interestingly, there was no similar gene pattern related to "red wine" consumption. In Figure 3, the CAIV factorial map shows the effects of beverages 1 h after intake. Samples which are grouped by beverage are represented by an ellipse. It can be seen that individuals drinking "alcohol" and "red wine" do not cluster together. According to the first discriminating axis they rather are separated in an opposite direction. It was found that several genes involved in "inflammatory response" are up-regulated after alcohol and down-regulated after red wine intake. Although, from these findings one can conclude that red wine might contain anti-inflammatory properties, it might represent a significant health hazard not tested in the current experiment (i.e. hepatic or neurologic toxicity).

### Comparison of CAIV with a gene-by-gene methods

Linear mixed-effect models were fitted to the data above. The factors "time" and "beverage" were defined as fixed factors, whereas the factor "individual" was defined as random. A 3-way ANOVA was performed and genes showing a significant beverage effect were extracted. A contrast alcohol vs. water and red wine vs. water was applied to our data in order to detect genes specifically dysregulated by the action of alcohol and red wine, taking water as control. Among the 100 most up-regulated genes after alcohol, we observed an enrichment of genes involved in "immune response" (GO:0006955, p-val < 0.001) and apoptosis (GO:0006915, p-val < 0.05). Regarding the effect of "red wine", genes identified by the gene-by-gene approach showed a poorer biological coherence compared to those found by using CAIV. In table 1, the enrichment of genes obtained by the two methods within 4 biologically relevant GO categories was compared. Results show that the level of significance of these enrichments is higher for CAIV in all the 4 categories. This analysis also indicates that CAIV may achieve a better "resolution" than ANOVA. The subcategory "I*κ*B kinase/NF*κ*B cascade", which is related to immune response and inflammation, is significantly enriched among genes extracted by CAIV (p-val = 0.02), whereas no genes of this category were identified by ANOVA. Overall, results show that CAIV selects genes with a higher biological coherence than ANOVA.

**Table 1:** GO analysis of genes obtained by CAIV compared to ANOVA.

GO categories	CAIV	ANOVA
"response to stimulus"	24% (p-val = 1.5E-2)	23% (p-val = 4E-2)
"immune response"	20% (p-val = 9.3E-6)	13% (p-val = 1.7E-2)
"apoptosis"	11% (p-val = 9.2E-4)	7% (p-val = 7.8E-2)
"I-*κ*B kinase/NF-*κ*B cascade"	4% (p-val = 2.6E-2)	0% (NS)

The GO analysis is applied to the 100 most discriminating/dysregulated genes specifically associated with the action of "red wine". Results are presented as the proportion of genes belonging to the GO category (%) and the enrichment significance (p-val).

## Discussion

### CAIV compared to other two-tables coupling method

RDA and CCA are two particular cases of analyses with respect to instrumental variables. Instrumental variables analyses can be associated with any single-table ordination techniques although CA is particularly efficient in microarray data analysis. CAIV is equivalent to CCA and orthogonal CAIV is equivalent to partial CCA.

Coinertia analysis is another method for linking two tables, and has been successfully applied to microarray data [16]. However in the case of coinertia analysis, the two tables are analysed symmetrically. Coinertia analysis explores the relationship between two statistical triplets, whereas instrumental variables analysis link one statistical triplet with a table of descriptive variables. The objective of coinertia analysis differs from instrumental variables techniques. Coinertia analysis is preferred when the number of explicative variables is high and it is less sensitive to correlated variables. Indeed, in contrast to instrumental variables analysis which links tables *Y *and *X *using multiple regression models, co-inertia analysis uses partial least square regression [17]. If the number of descriptive variables is low, then instrumental variables analysis is very efficient.

### Multivariate approaches vs. univariate approaches

For controlling sources of variations in microarray data, authors generally use univariate approaches. The significance of a gene dysregulation conditionally to one or several experimental variables is basically estimated by fitting linear models gene-by-gene. There are several limitations in the use of such a strategy. As genes are treated independently, one may loose the multi-dimensional information contained in the dataset. In addition, many constraints are associated with the use of ANOVA models. They include the normality assumption, the problems related to multiple testing and the cost of the replications needed when several factors are included in the analysis.

On the other hand, using a multivariate approach provides a means to take genes' co-variations and gene-gene interactions into account. Refinements of instrumental variables analysis and constrained ordinations were recently proposed. These methods include non-linear RDA and CCA [18], which extend the theoretical framework of the analyses with respect to instrumental variables to the non-linear modeling. It is noteworthy that CAIV as it is implemented in the package vegan (function cca) allows the modeling of interactions and contrasts.

One limitation of multivariate ordination methods is their exploratory nature. Like other constrained ordination methods, CAIV identifies axes that are best explained by a linear combination of descriptive variables. As such, CAIV can be used to select genes with high contribution to the modelled constraints. CAIV as it is presented in the present manuscript can be used to test the statistical significance of a constraint, but does not test the significance of individual gene contributions. The use of resampling methods including jackknifing and bootstraping for assessing the reliability and the stability of scores and loadings in multivariate analysis might provide a way of inferring the statistical significance of gene contributions [19,20].

## Conclusion

Analyses with respect to instrumental variables can easily be applied to microarray data for the exploration of complex data structures. They provide a convenient way to estimate the contribution of several factors. They can be used both to examine dimensions in the dataset and to remove confounding factors. In our example, relevant genes associated with a specific beverage were only unmasked after using an instrumental variables strategy. Although these methods are only exploratory, they can be used to explain trends and associations among samples and identify genes associated with specific factors.

Correspondence analysis with respect to instrumental variables is particularly appropriate for microarray data because CA better stresses sample-genes relationships [2,21], which eases the interpretation. This is also appropriate for data where the number of variables far exceeds the number of samples.

All these analyses have been implemented within the **R **package *ade4*. Many more multivariate techniques are proposed in this package together with some extensive graphical tools. Different other implementations of constrained ordination methods are also available in the **R **library *vegan*.

## Methods

### Statistical packages

All computations were performed using the statistical software **R **[22], the multivariate analysis package *ade4 *[23] and different packages from the Bioconductor project [24]. Monte-Carlo permutation test implemented both in the *ade4 *and *vegan *libraries were used to test the statistical significance of the instrumental variables analyses.

### Gene ontology

For the sake of biological interpretation, genes of interest were grouped according to Gene Ontology (GO) annotations [25]. The relevance of GO categories was assessed using enrichment tests implemented in the web tool DAVID [26].

## Authors' contributions

FB performed the analysis and wrote the paper. MHB, leader of the Pulmonary Gene Research group, supervised the work and gave input to the manuscript. MF and JW provided expertises regarding the biological relevance of our results. JS performed the microarray experiments.
